# Effectiveness of first-line antiretroviral therapy in the IPEC cohort, Rio de Janeiro, Brazil

**DOI:** 10.1186/1742-6405-11-29

**Published:** 2014-09-01

**Authors:** Sandra W Cardoso, Paula M Luz, Luciane Velasque, Thiago Torres, Lara Coelho, Kenneth A Freedberg, Valdilea G Veloso, Rochelle P Walensky, Beatriz Grinsztejn

**Affiliations:** 1 Instituto de Pesquisa Clínica Evandro Chagas, Fundação Oswaldo Cruz, Rio de Janeiro, Brasil; 2 Departamento de Matemática e Estatística, Universidade Federal do Estado do Rio de Janeiro, Rio de Janeiro, Brasil; 3 The Divisions of Infectious Disease and General Medicine, and the Medical Practice Evaluation Center, Department of Medicine, Massachusetts General Hospital and the Harvard University Center for AIDS Research, Harvard Medical School, Boston, MA, U.S.A

**Keywords:** HIV/AIDS, Antiretroviral treatment, Effectiveness, Cohort study, Rio de Janeiro, Brazil

## Abstract

**Background:**

While Brazil has had a long-standing policy of free access to antiretroviral therapy (ART) for all in need, the epidemiological impact of ART on human immunodeficiency virus (HIV) RNA suppression in this middle-income country has not been well evaluated. We estimate first-line ART effectiveness in a large Brazilian cohort and examine the socio-demographic, behavioral, clinical and structural factors associated with virologic suppression.

**Methods:**

Virologic suppression on first-line ART at 6, 12, and 24 months from start of ART was defined as having a viral load measurement ≤400 copies/mL without drug class modification and/or discontinuation. Drug class modification and/or discontinuation were defined based on the class of a particular drug. Quasi-Poisson regression was used to quantify the association of factors with virologic suppression.

**Results:**

From January 2000 through June 2010, 1311 patients started first-line ART; 987 (75%) patients used NNRTI-based regimens. Virologic suppression was achieved by 77%, 76% and 68% of patients at 6, 12 and 24 months, respectively. Factors associated with virologic suppression at 12 months were: >8 years of formal education (compared to <4 years, risk ratio (RR) 1.13, 95% confidence interval (95% CI) 1.03-1.24), starting ART in 2005-2010 (compared to 2000-2004, RR 1.25 95% CI 1.15-1.35), and clinical trial participation (compared to no participation, RR 1.08 95% CI 1.01-1.16). Also at 12 months, women showed less virologic suppression compared to heterosexual men (RR 0.90 95% CI 0.82-0.99). For the 24-month endpoint, in addition to higher education, starting ART in the later period, and clinical trial participation, older age and an NNRTI-based regimen were also independently associated with virologic suppression.

**Conclusions:**

Our results show that in Brazil, a middle-income country with free access to treatment, over three-quarters of patients receiving routine care reached virologic suppression on first-line ART by the end of the first year. Higher education, more recent ART initiation and clinical trial participation were associated with improved outcomes both for the 12-month and the 24-month endpoints, suggesting that further studies are needed to understand what aspects relating to these factors lead to higher virologic suppression.

## Background

Since the beginning of the HIV/AIDS epidemic, Brazil’s response has been both timely and inclusive, addressing prevention as well as treatment. A noteworthy moment was the decision to provide highly active antiretroviral therapy (ART) for all patients in need in 1996. With over 15 years of universal access to ART and almost 300,000 patients receiving ART, Brazil’s HIV treatment program stands alone in its universal coverage of all in need compared to other middle-income or resource-limited countries [1]. Despite the enormous publicity it has received, evaluations of Brazil’s HIV treatment program are limited. Studies have indicated that morbidity and mortality from HIV infection has fallen since the introduction of ART [2-4]. However, studies evaluating the impact of ART in suppressing HIV viral load, i.e. its effectiveness within the routine care provided through the public health system (the Unified Health System) of Brazil are scarce.

The efficacy of new drugs is assessed in short-term randomized clinical trials usually conducted in selected populations which frequently exclude participants with concurrent opportunistic diseases, substance abuse and/or psychiatric comorbidities [5]. As such, results from clinical trials are often not generalizable to all treated individuals who might be part of a clinical cohort or to longer-term outcomes [6]. Understanding ART effectiveness within the routine care setting is crucial to guide the evolution of the Brazilian HIV Treatment program. In this study, we evaluated first-line ART effectiveness for patients starting therapy from 2000 to 2010, as well as the factors that correlate with virologic suppression in a large urban cohort in Rio de Janeiro, Brazil.

## Methods

### The IPEC clinical cohort

This study was conducted at the Evandro Chagas Clinical Research Institute, Oswaldo Cruz Foundation (IPEC/FIOCRUZ), one of the largest infectious disease research centers in Brazil, where care has been provided to HIV/AIDS patients since the beginning of the AIDS epidemic in Brazil in 1986. An observational, longitudinal, clinical database is maintained on patients receiving primary and specialized outpatient and inpatient HIV care at the clinic; it includes socio-demographic, behavioral, clinical and therapeutic information. Details of the HIV/AIDS clinical cohort can be found elsewhere [7,8]. The IPEC Institutional Review Board reviewed and approved this study.

### Study population and definitions

All patients who started first-line ART between January 1, 2000 and June 30, 2010 were included and follow-up information included data through September 30, 2011. Though combination ART became available in mid-1996, we specifically excluded 1996-1999 because it was a period characterized by non-standardized combinations as well as continued exposure to mono or dual therapy, which were kept in the prior to 2000 guidelines as alternative first-line regimens. ART was defined as two nucleoside reverse transcriptase inhibitors (NRTI) in combination with one non-nucleoside reverse transcriptase inhibitor (NNRTI) or one protease inhibitor (PI). Drug class modification and/or discontinuation were defined based on the class of a particular drug. A patient who started a first-line NNRTI-based regimen, for example, was assumed to have modified and/or discontinued the regimen if it was changed to a PI-based regimen or if the NNRTI was discontinued. NRTI modifications and/or discontinuations were not considered since these could be due to reasons unrelated to a drug-class failure. For the years 2000-2010, ART guidelines were consistent regarding first-line ART with NNRTI-based regimens cited as preferred and PI-based regimens as alternative options.

### First-line ART effectiveness

First-line ART effectiveness was defined as having HIV viral load ≤ 400 copies/mL and no drug class modification and/or discontinuation. Deaths from AIDS-related causes were considered as failures. Because the limit of detection of viral load assays used throughout the study period varied from ≤400 copies/mL to ≤50 copies/mL, we used the ≤400 copies/mL threshold for the entire study period for consistency.

We examined virologic outcomes at 6, 12, and 24 months from first-line ART initiation. Window periods were defined for each time point as 5-9 months, 9-15 months, and 21-27 months, respectively. Within each window, the viral load measurement occurring closest to the target time point (either before or after) was chosen. Drug class modification and/or discontinuations were evaluated for the entire period from the start of first-line ART until the upper limit of each window period.

### Missing data

The IPEC Cohort has a validated algorithm for identification of deaths which has been previously described [8,9]. In addition, since IPEC provides outpatient and inpatient care, as well as a multidisciplinary team including a cadre of clinical specialties coupled with pharmaceutical care, the rate of loss-to-follow-up is low (4.1/100 person-years). Absence of laboratory measurements is most frequently a result of insufficient infrastructure to support the CD4/viral load monitoring needed for all patients on ART. As such, we evaluated the impact of missing viral load measurements on first-line ART effectiveness by examining both best and worst-case scenarios. In the best case scenario, missing viral load data were assumed as suppression. Alternatively, in the worst-case scenario, missing viral loads were imputed as failures.

### Immunologic response

We examined CD4 counts 6, 12, and 24 months from ART initiation. Window periods were, as for viral loads, defined for each time point as 5-9 months, 9-15 months, and 21-27 months. Within each window, the CD4 count occurring closest to the target time point (either before or after) was chosen.

### Statistical analyses

First-line ART effectiveness was calculated as the probability (95% CI) of viral suppression at 6, 12 and 24 months after ART initiation. The impact of socio-demographic, behavioral, clinical and structural factors on virologic suppression at 12 and 24 months was estimated using a quasi-Poisson regression model; this corrected for variance estimation and allowed for the estimation of relative risks. We chose the use of quasi-Poisson models since the data showed overdispersion (i.e. the variance was greater than the mean; this would be contrary to the assumption of the Poisson model which assumes that the variance is equal to the mean) and because log-binomial regression models did not converge. The final model included variables found to be significant at a threshold p-value of 0.05, as well as factors known to be clinically relevant or that were shown to modify the effect of a covariate in the adjusted model. We also examined the impact of missing viral load measurements on the final model by re-estimating the parameters while assuming the worst-case and best-case scenarios. R software version 2.15.2 (http://www.r-project.org) was used for all statistical analyses.

## Results

### Study population

From January 2000 through June 2010, 1,311 patients started first-line ART; 40% were ≥40 years old (Table 1). Among men, men who have sex with men (MSM) predominated as the HIV risk exposure category; 9.8% of the study population reported injection drug use (IDU) or other modes of HIV risk exposure. Half of the cohort had over 8 years of education. Sixty-four percent of the patients had three years or less since their first positive HIV test; 494 (37.7%) had a pre-treatment CD4 count ≤200 cells/μL (overall median 222/μL, IQR:105-322 cells/μL), and 466 (35.5%) had a pre-treatment viral load >100,000 copies/mL. The majority of patients started a first-line NNRTI-based regimen (987, 75.3%). Seventy percent of patients started their first-line regimens in the calendar period 2005-2010. Just over one-third of patients started first-line ART within a clinical trial conducted at IPEC.

**Table 1:** Baseline socio-demographic, behavioral, clinical and structural characteristics at first-line antiretroviral therapy (ART) initiation (IPEC Clinical Cohort, 2000 to 2010)

**Total**		**1311**
**Age**		
	Mean (SD)	37.1 (9.9)
	< 30	355 (27.1)
	30-39	446 (34.0)
	≥ 40	510 (38.9)
**Race**		
	Non-white	621 (47.4)
	White	690 (52.6)
**Gender/Risk category**^**a**^		
	Women	432 (33.0)
	Heterosexual men	327 (24.9)
	MSM	423 (32.3)
	IDU/Other	129 (9.8)
**Years of formal education**		
	< 4	277 (21.1)
	4-8	380 (29.0)
	> 8	654 (49.9)
**Years since HIV + test**		
	<= 3	836 (63.8)
	> 3	475 (36.2)
**Pre-treatment CD4 count/μL**^**b**^		
	Mean (SD)	233 (184)
	<= 200	494 (37.7)
	201-350	392 (29.9)
	> 350	208 (15.9)
	Missing	217 (16.6)
**Pre-treatment HIV viral load copies/mL**^**c**^		
	<= 100000	532 (40.6)
	> 100000	466 (35.5)
	Missing	313 (23.9)
**Concurrent AIDS defining illness**^**d**^		
	No	1013 (77.3)
	Yes	298 (22.7)
**Hepatitis B/C co-infection**^**e**^		
	No	1270 (96.9)
	Yes	41 (3.1)
**ART regimen**^**f**^		
	PI-based	324 (24.7)
	NNRTI-based	987 (75.3)
**Calendar year of ART initiation**		
	2000-2004	392 (29.9)
	2005-2009	919 (70.1)
**Started ART in clinical trial**		
	No	856 (65.3)
	Yes	455 (34.7)

SD: standard deviation, HIV: human immunodeficiency virus, PI: protease inhibitor, NNRTI: non-nucleoside reverse transcriptase inhibitor.

^a^Gender and reported mode of HIV exposure were categorized jointly into women, men who have sex with men (MSM), heterosexual men, and injection drug users (IDU, men and women) and other reported modes of HIV exposure (men and women). Individuals reporting both injection drug use and other modes of HIV exposure were categorized as IDU.

^b^Measurement closest to the date of start of ART up to 30 days after.

^c^Measurement closest to the date of start of ART up to 7 days after.

^d^Defined as the presence of any Centers for Disease Control and Prevention (CDC) 1993 disease from 90 days prior to up to 30 days after start of ART.

^e^Chronic Hepatitis B infection was defined as persistence of a positive HBsAg for more than six months without a subsequent negative HBsAg; chronic Hepatitis C infection was defined as a confirmed positive anti-HCV detected at least six months from the first test.

^f^Patients starting Integrase inhibitor-based regimens were too few and thus excluded (7 individuals with start of ART in 2010).

Unless otherwise stated, number (percentages) are shown.

Among the 987 patients who started an NNRTI-based regimen, efavirenz (EFV) was used by 93.3% (921/987); the most frequent EFV-based combination was zidovudine (AZT) + lamivudine (3TC) + EFV (590/921, 64.1%), followed by tenofovir (TDF) + 3TC + EFV or TDF + emtricitabine (FTC) + EFV (252/921, 27.4%). Among the 324 patients who started a PI-containing regimen, the majority used a boosted PI (197/324, 60.8%). The most frequent boosted PI was ritonavir-boosted lopinavir LPV/r (93/197, 47.2%) followed by ritonavir-boosted atazanavir ATV/r (62/197, 31.4%). Among those who started a non-boosted PI regimen (127/324, 39.2%), 65.4% (83/127) started with ATV. The frequency of boosted PI prescriptions was 47.8% (54/113) for the calendar year 2000-04 and 67.7% (143/211) for 2005-10.

### First-line ART effectiveness

Overall first-line ART effectiveness, inclusive only of those with viral load data, was 76.9%, 76.1% and 67.9% at 6, 12 and 24 months (Table 2, top section). When assuming the best-case scenario including all patients, ART effectiveness increased to 82.8%, 80.8% and 77.0% at 6, 12 and 24 months. Inclusive of all patients, the worse-case scenario produced ART effectiveness rates of 57.5%, 61.2% and 48.6% at 6, 12 and 24 months. First-line ART effectiveness at each time point was consistently higher for those using an NNRTI-based regimen compared to a PI-based regimen (Table 2, middle section). Increased effectiveness was also observed for those who started first-line ART in 2005-2010 compared to those who started in 2000-2004 (Table 2, bottom section).

**Table 2:** Effectiveness of first-line antiretroviral therapy at 6, 12, and 24 months from start of antiretroviral therapy (ART) stratified by regimen and calendar year

		**6 months**	**12 months**	**24 months**
**Overall**		**N (%)**	**N (%)**	**N (%)**
	For patients with HIV VL	754/980 (76.9)	802/1054 (76.1)	637/938 (67.9)
	Best-case scenario^a^	1085/1311 (82.8)	1059/1311 (80.8)	1010/1311 (77.0)
	Worst-case scenario^b^	754/1311 (57.5)	802/1311 (61.2)	637/1311 (48.6)
**Stratified by type of ART regimen**				
**NNRTI-based**				
	For patients with HIV VL	583/740 (78.8)	603/784 (76.9)	503/711 (70.7)
	Best-case scenario	830/987 (84.1)	806/987 (81.7)	779/987 (78.9)
	Worst-case scenario	583/987 (59.1)	603/987 (61.1)	503/987 (51.0)
**PI-based**				
	For patients with HIV VL	171/240 (71.3)	199/270 (73.7)	134/227 (59.0)
	Best-case scenario	255/324 (78.7)	253/324 (78.1)	231/324 (71.3)
	Worst-case scenario	171/324 (52.8)	199/324 (61.4)	134/324 (41.4)
**Stratified by calendar year of ART initiation**				
**2000-2004**				
	For patients with HIV VL	157/252 (62.3)	188/297 (63.3)	171/287 (59.6)
	Best-case scenario	297/392 (75.8)	283/392 (72.2)	276/392 (70.4)
	Worst-case scenario	157/392 (40.1)	188/392 (48.0)	171/392 (43.6)
**2005-2010**				
	For patients with HIV VL	597/728 (82.0)	614/757 (81.1)	466/651 (71.6)
	Best-case scenario	788/919 (85.7)	776/919 (84.4)	734/919 (79.9)
	Worst-case scenario	597/919 (65.0)	614/919 (66.8)	466/919 (50.7)

HIV: human immunodeficiency virus, VL: viral load, PI: protease inhibitor, NNRTI: non-nucleoside reverse transcriptase inhibitor.

^a^Best-case scenario assumes missing viral load data as suppression.

^b^Worst-case scenario assumes missing viral loads data as failures.

### CD4 counts

**Figure 1:**
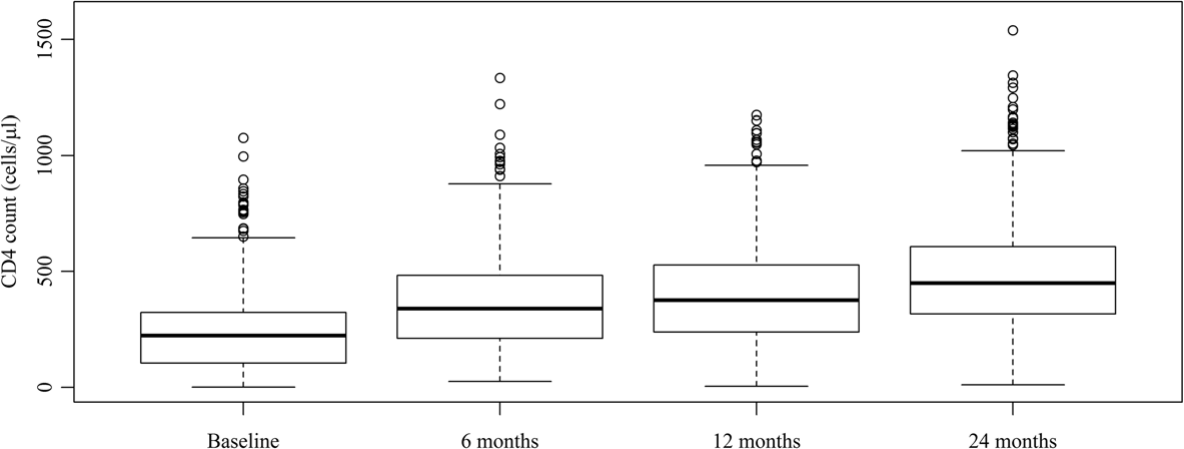
**Box plot of CD4 count distribution at baseline and 6, 12, and 24 months after antiretroviral therapy initiation.** Pair-wise comparisons of baseline measurement with 6-, 12-, and 24-month time points indicate statistically significant differences (Wilcoxon paired test).

Median CD4 counts for the entire cohort significantly increased with the progression of the time points evaluated (Figure 1). At baseline, the median CD4 count was 221/μL whereas at 6, 12 and 24 months, it was 338/μL, 375/μL and 448/μL. These improvements correspond to a median CD4 count increase from baseline of 107/μL, 151/μL and 242/μL at 6, 12, and 24 months.

### Factors associated with virologic suppression

In the adjusted model for the 12-month endpoint, gender/risk category, education, calendar year of ART initiation and participation in a clinical trial all remained independently associated with virologic suppression (Table 3). Compared to heterosexual men, women were less likely to be virologically suppressed (RR 0.90 95% CI 0.82-0.99). Having over eight years of formal education resulted in improved virologic suppression (RR 1.13 95% CI 1.03-1.24), compared to less than four years of formal education. Increased virologic suppression was also associated with starting ART in 2005-2010 (RR 1.25 95% CI 1.15-1.35) compared to starting in 2000-2004. Participation in a clinical trial versus not was associated with increased virologic suppression (RR 1.08 95% CI 1.01-1.16).

**Table 3:** Unadjusted and adjusted relative risks (95% confidence intervals) for first-line antiretroviral therapy effectiveness at 12 and 24 months (IPEC cohort, 2000 to 2010)

		**12 months**		**24 months**	
		**Unadjusted**	**Adjusted**	**Unadjusted**	**Adjusted**
		**RR (95% CI)**	**RR (95% CI)**	**RR (95% CI)**	**RR (95% CI)**
**Age**					
	< 30	Ref.		Ref.	Ref.
	30-39	0.99 (0.91, 1.08)		1.05 (0.93, 1.18)	1.06 (0.94, 1.19)
	> = 40	1.04 (0.95, 1.13)		1.09 (0.98, 1.22)	**1.12 (1.00, 1.26)**
**Race**					
	Non-white	Ref.		Ref.	
	White	**1.07 (1.00, 1.14)**		1.05 (0.97, 1.15)	
**Gender/Risk category**^**a**^					
	Women	**0.89 (0.82, 0.98)**	**0.90 (0.82, 0.99)**	0.92 (0.82, 1.03)	0.94 (0.84, 1.06)
	Heterosexual men	Ref.	Ref.	Ref.	Ref.
	MSM	1.05 (0.97, 1.15)	1.02 (0.93, 1.12)	1.11 (0.99, 1.25)	1.11 (0.98, 1.25)
	IDU/Other	1.02 (0.90, 1.16)	1.02 (0.89, 1.16)	0.93 (0.78, 1.11)	0.95 (0.79, 1.13)
**Education**					
	< 4 years	Ref.	Ref.	Ref.	Ref.
	4-8 years	1.06 (0.96, 1.17)	1.07 (0.96, 1.18)	1.12 (0.98, 1.28)	1.13 (0.99, 1.29)
	> 8 years	**1.16 (1.06, 1.27)**	**1.13 (1.03, 1.24)**	**1.17 (1.04, 1.32)**	**1.14 (1.00, 1.29)**
**Years since HIV + test**					
	<= 3	Ref.		Ref.	
	> 3	0.97 (0.90, 1.04)		0.94 (0.86, 1.03)	
**Baseline CD4 cell count**^**b**^					
	<= 200	Ref.		Ref.	
	201-350	1.05 (0.97, 1.14)		1.01 (0.91, 1.12)	
	> 350	1.09 (0.99, 1.20)		1.11 (0.97, 1.26)	
	Missing	0.92 (0.83, 1.03)		1.01 (0.88, 1.15)	
**Baseline HIV viral load**^**c**^					
	<= 100000	Ref.		Ref.	
	> 100000	1.00 (0.93, 1.08)		1.03 (0.93, 1.14)	
	Missing	0.95 (0.87, 1.04)		0.99 (0.88, 1.11)	
**Concurrent ADI**^**d**^					
	No	Ref.		Ref.	
	Yes	1.06 (0.98, 1.16)		1.06 (0.95, 1.18)	
**Hepatitis B/C co-infection**^**e**^					
	No	Ref.		Ref.	
	Yes	1.16 (0.95, 1.43)		1.07 (0.83, 1.39)	
**Initial ART regimen**^**f**^					
	PI-based	Ref.		Ref.	Ref.
	NNRTI-based	1.04 (0.96, 1.13)		**1.20 (1.08, 1.34)**	**1.17 (1.05, 1.31)**
**Calendar year of ART initiation**					
	2005-2010	**1.28 (1.18, 1.39)**	**1.25 (1.15, 1.35)**	**1.20 (1.09, 1.33)**	**1.14 (1.03, 1.27)**
	2000-2004	Ref.	Ref.	Ref.	Ref.
**Started ART in clinical trial**					
	No	Ref.	Ref.	Ref.	Ref.
	Yes	**1.10 (1.03, 1.18)**	**1.08 (1.01, 1.16)**	**1.14 (1.04, 1.25)**	**1.12 (1.02, 1.23)**

Bold numbers indicate statistically significant results.

HIV: human immunodeficiency virus, VL: viral load, PI: protease inhibitor, NNRTI: non-nucleoside reverse transcriptase inhibitor.

^a^Gender and reported mode of HIV exposure were categorized jointly into women, men who have sex with men (MSM), heterosexual men, and injection drug users (IDU, men and women) and other reported modes of HIV exposure (men and women). Individuals reporting both injection drug use and other modes of HIV exposure were categorized into IDU.

^b^Measurement closest to the date of start of ART up to 30 days after.

^c^Measurement closest to the date of start of ART up to 7 days after.

^d^Defined as the presence of any Centers for Disease Control and Prevention (CDC) 1993 ADI at 90 days prior to up to 30 days after start of ART.

^e^Chronic Hepatitis B infection was defined as persistence of a positive HBsAg for more than six months without a subsequent negative HBsAg; chronic Hepatitis C infection was defined as a confirmed positive anti-HCV detected at least six months from the first test.

^f^Patients starting Integrase inhibitor-based regimens were too few and thus excluded (7 individuals with start of ART in 2010).

In the adjusted model for the 24-month endpoint, age, gender/risk category, education, type of ART regimen, calendar year of ART initiation, and clinical trial participation remained independently associated with virologic suppression (Table 3). Virologic suppression was associated with older age (RR for ≥40 years old 1.12 95% CI 1.00-1.26, compared to < 30 years old), and there was a trend toward greater virologic suppression among MSM compared to heterosexual men (RR 1.11 95% CI 0.98-1.27). Again, women compared to heterosexual men were less likely to be virologically suppressed, although this estimate did not reach statistical significance. Virologic suppression remained associated with first-line NNTRI-based regimen (RR 1.17 95% CI 1.05-1.31, compared to PI-based regimen), calendar year 2005-10 (RR 1.14 95% CI 1.03-1.27, compared to 2000-2004), and participation in a clinical trial (RR 1.12 95% CI 1.02-1.23).

The impact of missing viral load measurements on the adjusted models (Table 4) showed that the direction of the association of each variable with the 12-month and the 24-month outcomes remained the same for both the best case and worst case scenarios.

**Table 4:** Adjusted relative risks (95% confidence intervals) for first-line antiretroviral (ART) effectiveness at 12 and 24 months when assuming best-case (missing as success) and worst-case scenarios (missing as failure) (IPEC cohort, 2000 to 2010)

		**12 months**			**24 months**		
		**RR (95% CI)**	**RR (95% CI)**	**RR (95% CI)**	**RR (95% CI)**	**RR (95% CI)**	**RR (95% CI)**
		**Missing excluded**	**Missing = Success**	**Missing = Failure**	**Missing excluded**	**Missing = Success**	**Missing = Failure**
**Age**							
	< 30				Ref.	Ref.	Ref.
	30-39				1.06 (0.94, 1.19)	1.01 (0.94, 1.09)	1.15 (0.99, 1.33)
	> = 40				1.12 (1.00, 1.26)	1.04 (0.97, 1.13)	1.24 (1.07, 1.44)
**Gender/Risk category**							
	Women	0.90 (0.82, 0.99)	0.93 (0.87, 1.00)	0.87 (0.77, 0.98)	0.94 (0.84, 1.06)	0.94 (0.87, 1.02)	0.98 (0.84, 1.14)
	Heterosexual men	Ref.	Ref.	Ref.	Ref.	Ref.	Ref.
	MSM	1.02 (0.93, 1.12)	1.03 (0.96, 1.11)	0.98 (0.87, 1.10)	1.11 (0.98, 1.25)	1.08 (0.99, 1.17)	1.05 (0.90, 1.22)
	IDU/Other	1.02 (0.89, 1.16)	1.03 (0.94, 1.14)	0.89 (0.75, 1.05)	0.95 (0.79, 1.13)	0.97 (0.87, 1.09)	0.87 (0.70, 1.09)
**Education**							
	< 4 years	Ref.	Ref.	Ref.	Ref.	Ref.	Ref.
	4-8 years	1.07 (0.96, 1.18)	1.05 (0.97, 1.13)	1.05 (0.93, 1.20)	1.13 (0.99, 1.29)	1.06 (0.98, 1.16)	1.18 (1.00, 1.39)
	> 8 years	1.13 (1.03, 1.24)	1.08 (1.01, 1.17)	1.16 (1.03, 1.30)	1.14 (1.00, 1.29)	1.07 (0.99, 1.16)	1.21 (1.03, 1.41)
**Initial ART regimen**							
	PI-based				Ref.	Ref.	Ref.
	NNRTI-based				1.17 (1.05, 1.31)	1.09 (1.02, 1.17)	1.21 (1.05, 1.39)
**Calendar year of ART initiation**							
	2005-2009	1.25 (1.15, 1.35)	1.14 (1.08, 1.22)	1.34 (1.21, 1.49)	1.14 (1.03, 1.27)	1.11 (1.03, 1.19)	1.08 (0.95, 1.23)
	2000-2004	Ref.	Ref.	Ref.	Ref.	Ref.	Ref.
**Started ART in clinical trial**							
	No	Ref.	Ref.	Ref.	Ref.	Ref.	Ref.
	Yes	1.08 (1.01, 1.16)	1.05 (0.99, 1.11)	1.10 (1.00, 1.21)	1.12 (1.02, 1.23)	1.04 (0.98, 1.11)	1.24 (1.10, 1.39)

## Discussion

In this large cohort study of HIV-infected patients in Rio de Janeiro, Brazil, we estimated ART effectiveness for patients cared for between 2000 and 2010. We found that ART effectiveness at 6 months was 77% among patients with viral load results and no drug class modification and/or discontinuation. Using an intent-to-continue-treatment approach, that is, a less stringent criterion compared to that of the present study, the ART-CC cohort reported an estimate of 76% of undetectable viral load 6 months after ART initiation [10]. For the 12-month time point, our estimate of 61% for ART effectiveness when assuming missing data equals failure is consistent with that reported in a systematic review of clinical trials and cohort studies that employed the same approach to evaluating first-line ART efficacy (57-66% [11-13]). In line with our results, Barth et al., in a study conducted in rural South Africa, found 55% effectiveness using the same intent-to-treat approach [14]. It is important to consider the calendar period, the stringency of study definitions and the availability of one pill once-daily regimens, all factors that could partially explain diverging suppression rates in other cohort studies when compared to our findings [5,6,10,11,13-23].

We showed in a stratified analysis that ART effectiveness was higher for NNRTI-based regimens. These findings corroborate results from clinical trials and cohort studies that demonstrate greater effectiveness of NNRTI-based regimens, in particular, of efavirenz-based regimens [11,19,24]. In the adjusted analysis, however, the NNRTI-based regimen was found to be independently associated with virologic suppression solely at the 24-month endpoint; as such, when other factors were taken into account the regimens were not significantly different. LPV/r was the most frequently prescribed PI, as recommended by the Brazilian HIV Treatment Guidelines, which may explain the poorer outcomes observed with PI-based regimens, as opposed to the comparable effectiveness shown in AIDS Clinical Trials Group (ACTG5202), when boosted atazanavir was the PI comparator [25]. Of note, 39% of the patients in our cohort who started a PI-based regimen started on a non-boosted PI, of which 65% were atazanavir-based. It is well known that drug regimens including non-boosted PIs have poorer outcomes compared to other ART strategies [26]. The ACTG A5175 PEARLS trial, which was conducted in both high-income and low-middle-income settings, found non-boosted atazanavir to be inferior to efavirenz-based regimens [27]. Thus, the use of non-boosted PI-based regimens could partially explain the differences in ART effectiveness between our study and these trial findings.

Our study covered a time span of 11 years which allowed us to evaluate ART effectiveness in two periods, namely 2000-2004 and 2005-2010. Both the crude estimates and the adjusted analyses showed that the more recent calendar period was associated with increased ART effectiveness. This finding most likely results from the availability, more recently, of regimens with improved drug combinations, drugs with better tolerability and dosing convenience and, as a result, improved treatment adherence [28].

We found that clinical trial participation was independently associated with virologic suppression at 12 and 24 months from start of ART, corroborating other published results [29,30]. Notably, in contrast to routine care, clinical trial participants are more intensively followed, adherence to study visits and drugs is monitored, drug toxicities are closely sought, and access to medical appointments is facilitated. For the time points evaluated in this study, clinical trial participants had significantly fewer missing viral load measurements. We believe these results highlight the need for more vigilant monitoring within the routine care provided by Brazil’s Unified Health System in order to improve ART effectiveness. Additionally, a comparative analysis of the procedures carried out in routine care versus the clinical trial setting could shed light into the most important aspects of trial participation that lead to increased ART effectiveness. Further studies are also needed to evaluate the long-term benefits of clinical trial participation, given that at the end of the trials patients are fully incorporated into routine care.

Two socio-demographic factors (older age and higher education level) were found to be independently associated with increased ART effectiveness, and there was a trend toward increased virologic suppression for one behavioral factor (MSM HIV risk exposure). Regarding older age, our findings corroborate results from other cohort studies that have found increased ART effectiveness among older individuals [31,32]. Likewise, improved virologic response among those with more years of formal education has also been reported in studies from both Brazil and the United States [33,34]. Older age and higher education are likely correlated with a better understanding of the importance and value of ART and, consequently, better treatment adherence [35,36]. We also found that MSM, compared to heterosexual men, had increased ART effectiveness. In our study population, MSM was linked to higher education, as 69% of the MSM reported > 8 years of formal education while only 38% of the women and heterosexual men reported this same level of education. In the multivariate model for the 12-month endpoint, women were found to have decreased ART effectiveness. In other studies that considered ART discontinuations as failures, men showed improved ART outcomes when compared to women [37]. Moreover, several clinical trials [38] and observational studies [7,39,40] have described a higher frequency of ART-related adverse events among women compared to men. In our cohort, we have previously reported that the hazard of ART modification or discontinuation for women is 1.67 times the hazard for men within the first year of treatment [7]. For Brazil, these findings highlight the need to focus interventions aiming to improve ART outcomes among young, less educated heterosexual men and women, and to address specific issues particularly among women including ART tolerability and competing caretaking priorities.

Our study has several limitations. One is the substantial fraction of missing viral load measurements. We addressed this limitation by conducting sensitivity analyses which allowed us to generate upper and lower limits for the ART effectiveness estimates. We also evaluated the impact of the missing viral loads on the adjusted analysis by modeling both best- and worst-case scenarios. These modeling exercises generated results which are similar to those obtained when the missing data were excluded. In contrast, CD4 counts were not imputed and did suffer from a somewhat smaller degree of missing data, and therefore care is needed when extrapolating from these results.

In summary, we have shown that in Brazil, a middle-income country universal access to care and treatment, virologic suppression on first-line ART was achieved by over three-quarters of patients receiving routine care in a public facility. We also studied factors associated with virologic suppression at 12- and 24 months since ART initiation and found that higher education, more recent ART initiation and clinical trial participation were associated with improved outcomes. To translate these findings into applicable interventions to improve ART outcomes, the specifics relating to the factors leading to higher virologic suppression need to be further studied.

## Competing interests

The authors declare no conflict of interest.

## Authors’ contributions

SWC contributed to the conception and design of the study, acquired the data, and drafted the manuscript. PML contributed to the conception and design of the study, and drafted the manuscript. LV contributed to the conception and design of the study, and performed the statistical analysis. TT and LC acquired and revised the data. KAF participated in the study’s design, discussed the results, helped to draft and revise the manuscript for important intellectual content. VGV participated in the study’s design and coordination and helped to draft the manuscript. RPW participated in the study’s design, discussed the results, helped to draft and revise the manuscript for important intellectual content. BG contributed to the conception and design of the study, coordinated efforts, drafted and revised the manuscript for important intellectual content. All authors read and approved the final manuscript.
